# Crystal structures and Hirshfeld surface analyses of 4,4′-{[1,3-phenyl­enebis(methyl­ene)]bis­(­oxy)}bis­(3-meth­oxy­benzaldehyde) and 4,4′-{[(1,4-phenyl­ene­bis(methyl­ene)]bis­(­oxy)}bis­(3-meth­oxy­benzalde­hyde)

**DOI:** 10.1107/S2056989019006662

**Published:** 2019-05-24

**Authors:** Saleem Iqbal, Vijayan Viswanathan, Devadasan Velmurugan, Tamilselvan Abiraman, Sengottuvelan Balasubramanian, Krishnasamy Gunasekaran

**Affiliations:** a Centre of Advanced Study in Crystallography and Biophysics, University of Madras, Guindy Campus, Chennai 600 025, India; b Department of Biophysics, All India Institute of Medical Sciences, New Delhi 110 029, India; c Department of Inorganic Chemistry, University of Madras, Guindy Campus, Chennai 600 025, India

**Keywords:** crystal structure, vanillin, 4-hy­droxy-3-meth­oxy­benzaldehyde, hydrogen bonding, C—H⋯O hydrogen bonds, C—H⋯π inter­actions, supra­molecular framework, Hirshfeld surface analysis, fingerprint plots

## Abstract

The title compounds, C_24_H_22_O_6_ (I) and C_24_H_22_O_6_ (II), each crystallize with half a mol­ecule in the asymmetric unit. The whole mol­ecule of compound (I) is generated by twofold rotation symmetry, the twofold axis bis­ecting the central benzene ring. The whole mol­ecule of compound (II) is generated by inversion symmetry, the central benzene ring being located on an inversion center. In (I), the outer benzene rings are inclined to each other by 59.96 (10)° and by 36.74 (9)° to the central benzene ring. The corresponding dihedral angles in (II) are 0.0 and 89.87 (12)°. In the crystal of (I), mol­ecules are linked by C—H⋯O hydrogen bonds and C—H⋯π inter­actions, forming ribbons propagating along the [10] direction. In the crystal of (II), mol­ecules are linked by C—H⋯O hydrogen bonds, forming a supra­molecular framework. The Hirshfeld surface analyses indicate that for both compounds the H⋯H contacts are the most significant, followed by O⋯H/H⋯O and C⋯H/H⋯C contacts.

## Abstract 2

The title compounds, 4,4′-{[1,3-phenyl­enebis(methyl­ene)]bis­(­oxy)}bis­(3-meth­oxy­benzaldehyde) (I) and 4,4′-{[(1,4-phenyl­enebis(methyl­ene)]bis­(­oxy)}bis­(3-meth­oxy­benzaldehyde) (II), each crystallize with half a mol­ecule in the asymmetric unit. The whole mol­ecule of compound (I) is generated by twofold rotation symmetry, while the whole mol­ecule of compound (II) is generated by inversion symmetry.

## Chemical context   

Vanillin, a phenolic compound, has been reported to offer neuroprotection against experimental Huntington’s disease and global ischemia by virtue of its anti­oxidant, anti-inflammatory and anti­apoptotic properties. Vanillin is a potential future therapeutic agent by virtue of its multiple pharmacological properties relevant to the treatment of neurodegenerative diseases (Dhanalakshmi *et al.*, 2015 ▸). Structural elements of vanillin have been observed to show anti­fungal activity (Fitzgerald *et al.*, 2005 ▸). Studies have revealed that the root and pod extracts of the plants *Heiidesmus Indicus* and *vanilla planifola* (plant-based food-flavouring agents) produce the fragrant phenolic compounds 2-hy­droxy-4-meth­oxy­benz­aldehyde (MBALD) and 4-hy­droxy-3-meth­oxy­benzaldehyde (vanillin). These compounds have been shown to be effective in treating Alzheimer’s disease and other neurological dysfunctions (Kundu & Mitra, 2013 ▸). Vanillin derivatives with various homocyclic or heterocyclic and hydro­phobic or hydro­philic moieties have shown tyrosinase inhibitory activity (Ashraf *et al.*, 2015 ▸). In view of the inter­est in such compounds we have synthesized 4,4′-{[1,3-phenyl­enebis(methyl­ene)]bis(oxy)}bis­(3-meth­oxy­benzaldehyde) (I) and 4,4′-{[(1,4-phenyl­ene­bis(methyl­ene)]bis­(­oxy)}bis­(3-meth­oxy­benzaldehyde) (II), and report herein on their crystal structures and Hirshfeld surface analyses.
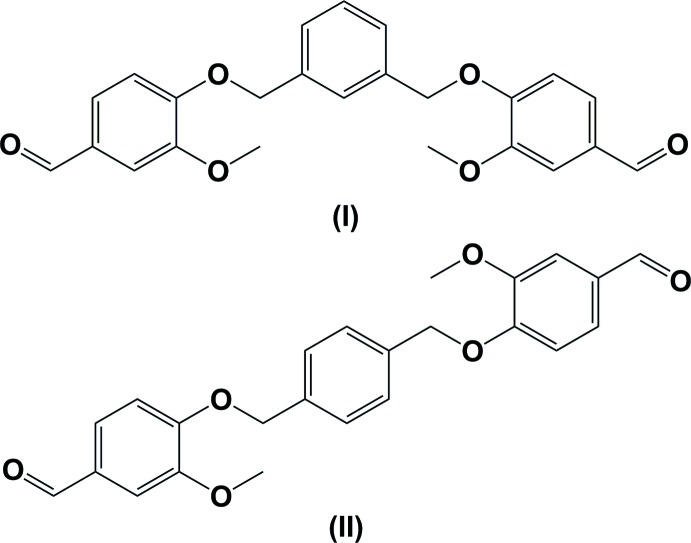


## Structural commentary   

**Figure 1:**
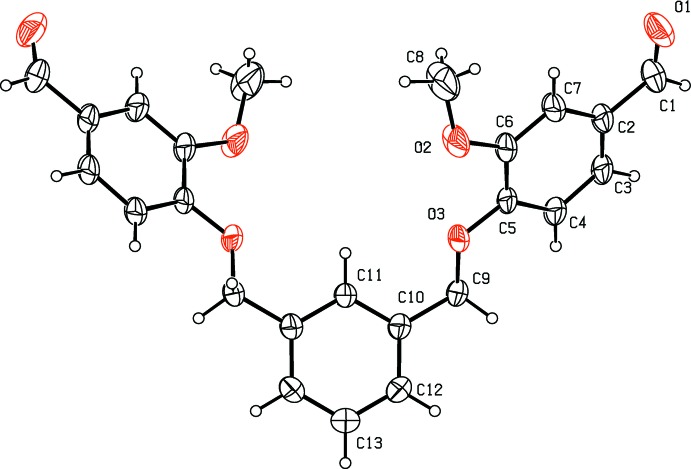
The mol­ecular structure of compound (I), with atom labelling (unlabelled atoms are related to labelled atoms by the symmetry operation −*x* + 2, *y*, −*z* + 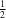). Displacement ellipsoids are drawn at 30% probability level.

The mol­ecular structure of compound (I) is shown in Fig. 1 ▸. The asymmetric unit consists of half a mol­ecule, the other half being generated by twofold rotation symmetry; the twofold axis bis­ects atoms C11 and C13 of the central benzene ring. The dihedral angle between the central benzene ring (C10–C13/C10′/C12′) and the outer benzene ring (C2–C7/C2′–C7′) is 36.74 (9)° [symmetry code: (’) −*x* + 2, *y*, −*z* + 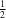). The outer benzene rings are inclined to each other by 59.96 (10)°. The acetaldehyde and meth­oxy­methane groups adopt extended conformations, as can be seen from the torsion angles C3—C2—C1—O1 = 180.0 (3)° and C5—C6—O2—C8 = −160.7 (3)°. Atoms C1 and O1 deviate from the plane of the benzene ring by 0.021 (2) and 0.034 (2) Å, respectively, while atoms O2 and C8 deviate from the plane of the benzene ring by −0.032 (2) and −0.471 (4)Å, respectively.

**Figure 2:**
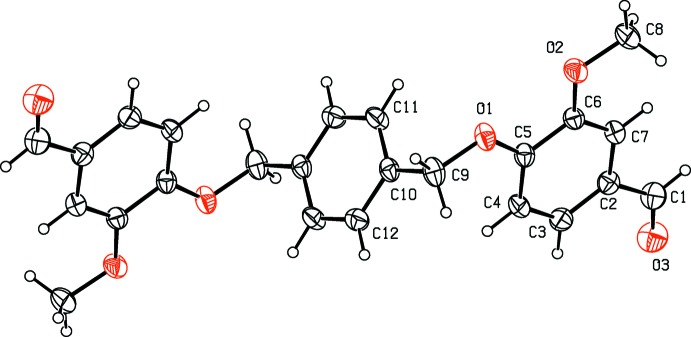
The mol­ecular structure of compound (II), with atom labelling (unlabelled atoms are related to labelled atoms by the symmetry operation −*x* + 1, −*y* + 1, −*z* + 1). Displacement ellipsoids are drawn at 30% probability level.

The mol­ecular structure of compound (II) is shown in Fig. 2 ▸. The asymmetric unit consists of half a mol­ecule, the other half being generated by inversion symmetry; the central benzene ring being situated about the inversion center. The outer benzene rings are parallel to each other and normal to the central benzene ring with a dihedral angle of 89.87 (12)°. The meth­oxy­methane and acetaldehyde groups adopt extended conformations, as can be seen from the torsion angles C5—C6—O2—C8 = 172.7 (2) Å and C7—C2—C1—O3 = −178.5 (3)°. Here, atoms O2 and C8 deviate from the plane of the benzene ring by −0.025 (2) and −0.211 (4) Å, respectively, while atoms C1 and O1 deviate from the plane of the benzene ring by 0.023 (3) and 0.056 (2) Å, respectively.

## Supra­molecular features   

**Figure 3:**
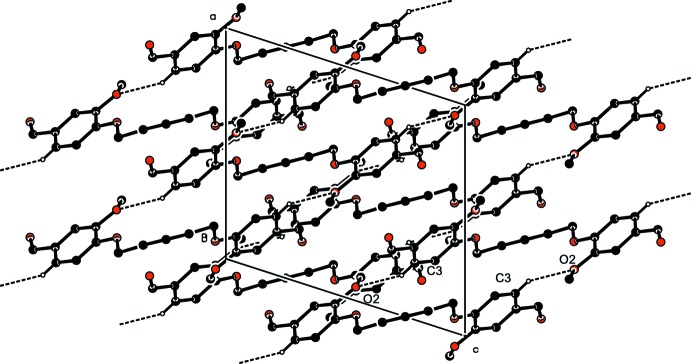
The crystal packing of compound (I), viewed along the *b* axis. The C—H⋯O hydrogen bonds (Table 1 ▸) are shown as dashed lines. For clarity, only the hydrogen atoms involved in hydrogen bonding have been included.

**Figure 4:**
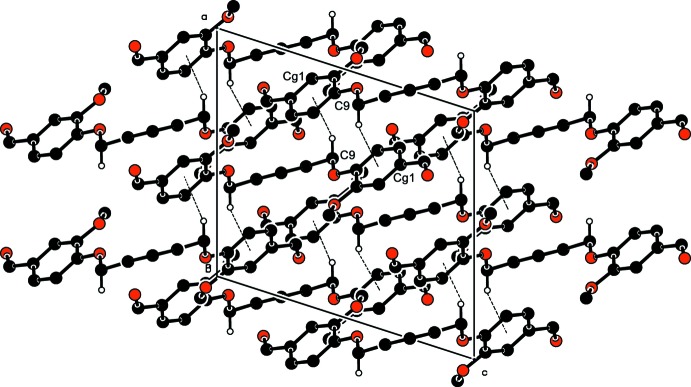
The crystal packing of compound (I), viewed along the *b* axis. The C—H⋯π inter­actions (Table 1 ▸) are shown as dashed lines. For clarity, only the hydrogen atoms involved in these inter­actions have been included.

In the crystal of (I), mol­ecules are linked by C3—H3⋯O^i^ hydrogen bonds forming ribbons propagating along the [10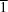] direction (Table 1 ▸ and Fig. 3 ▸). Within the ribbons mol­ecules are also linked by C—H⋯π inter­actions (Table 1 ▸), as shown in Fig. 4 ▸.

**Figure 5:**
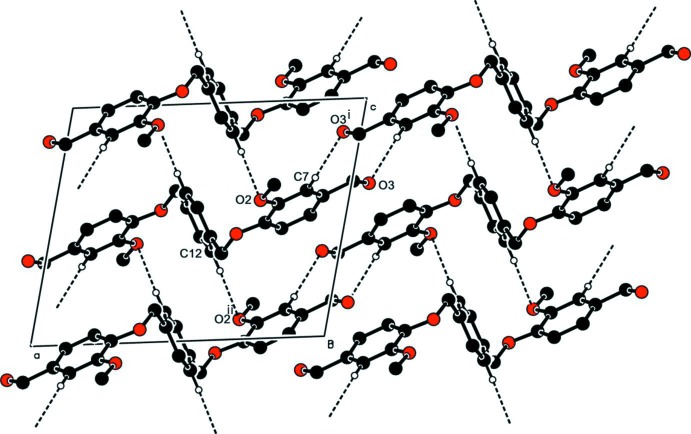
The crystal packing of compound (II), viewed along the *b* axis. the C—H⋯O hydrogen bonds (Table 2 ▸) are shown as dashed lines. For clarity, only the hydrogen atoms involved in hydrogen bonding have been included.

In the crystal of (II), mol­ecules are linked by C7—H7⋯O3^i^ and C12—H12⋯O2^ii^ hydrogen bonds (Table 2 ▸), forming a supra­molecular framework, as shown in Fig. 5 ▸.

## Hirshfeld surface analysis   

The Hirshfeld surface analysis (Spackman & Jayatilaka, 2009 ▸) and the associated two-dimensional fingerprint plots (McKinnon *et al.*, 2007 ▸) were performed using *CrystalExplorer17* (Turner *et al.*, 2017 ▸).

**Figure 6:**
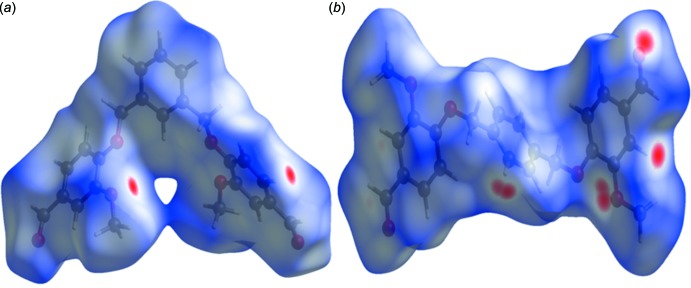
The Hirshfeld surface mapped over *d*_norm_, for (*a*) compound (I) and (*b*) compound (II).

**Figure 7:**
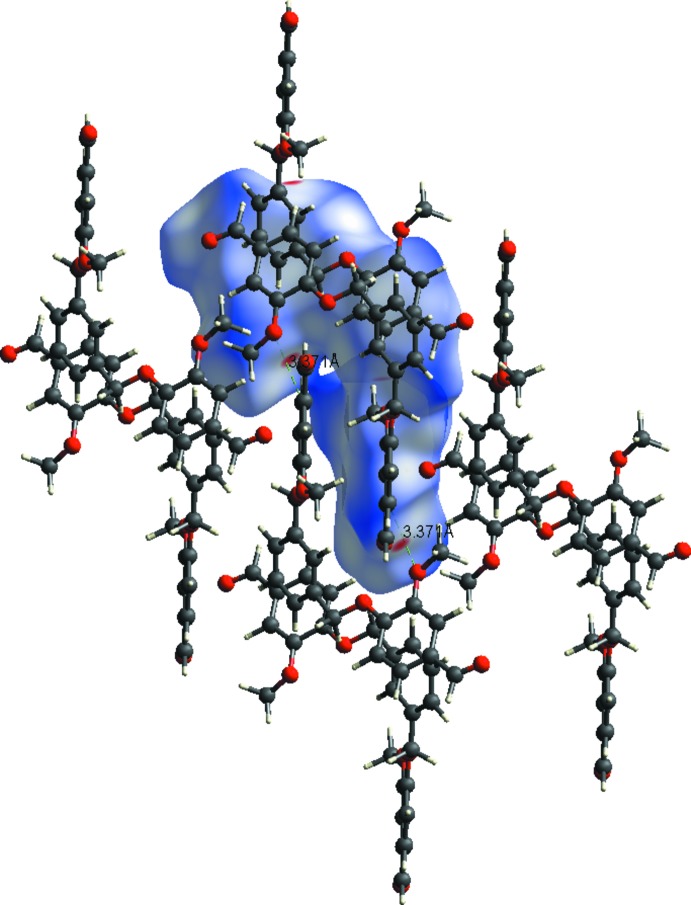
A view of the Hirshfeld surface mapped over *d*_norm_ for compound (I), showing the various inter­molecular contacts in the crystal.

The Hirshfeld surfaces of compounds (I) and (II) mapped over *d*_norm_ are given in Fig. 6 ▸ *a* and 6*b*, respectively. Views of the inter­molecular contacts in the crystals are shown in Figs. 7 ▸ and 8 ▸, for compounds (I) and (II), respectively. They are colour-mapped with the normalized contact distance, *d*_norm_, from red (distances shorter than the sum of the van der Waals radii) through white to blue (distances longer than the sum of the van der Waals radii). The blue region represents the positive electrostatic potential over the surface. The *d*_norm_ surface was mapped over a colour scale in arbitrary units of −0.156 (red) to 1.705 (blue) for compound (I) and −0.207 (red) to 1.206 (blue) for compound (II), where the red spots indicate the inter­molecular contacts involved in the hydrogen bonding.

**Figure 9:**
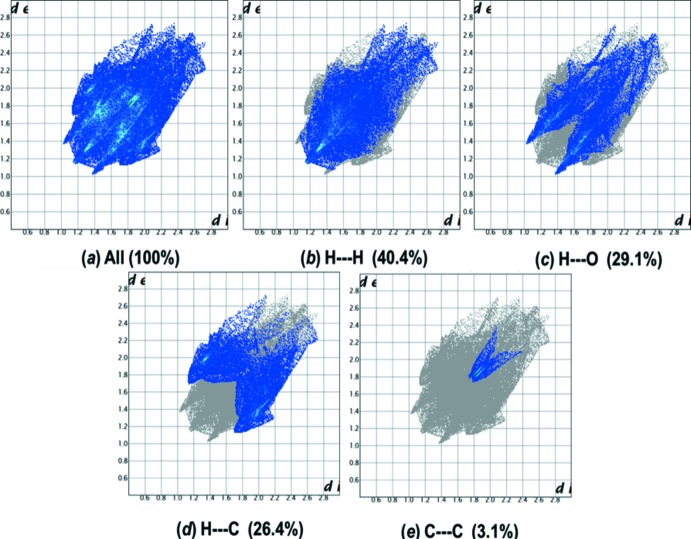
(*a*) The two-dimensional fingerprint plot for compound (I), and the fingerprint plots delineated into (*b*) H⋯H, (*c*) O⋯H/H⋯O, (*d*) C⋯H/H⋯C and (*e*) C⋯C contacts.

**Figure 10:**
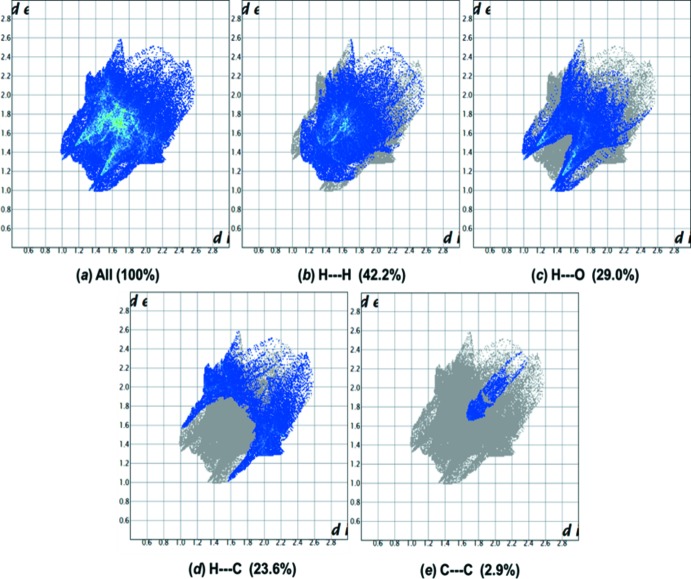
(*a*) The two-dimensional fingerprint plot for compound (II), and fingerprint plots delineated into (*b*) H⋯H, (*c*) O⋯H/H⋯O, (*d*) C⋯H/H⋯C and (*e*) C⋯C contacts.

The two-dimensional fingerprint plots [Fig. 9 ▸ for (I) and Fig. 10 ▸ for (II)] are deconvoluted to highlight atom-pair close contacts by which different atomic types, overlapping the full fingerprint plot can be separated based on different inter­action types. For compound (I), inter­molecular H⋯H contacts of 40.4% (Fig. 9 ▸ *b*) are the most significant, followed by 29.1% for O⋯H/H⋯O (Fig. 9 ▸ *c*), 26.4% for C⋯H/H⋯C (Fig. 9 ▸ *d*) and 3.1% for C⋯C (Fig. 9 ▸ *e*) contacts. In contrast, for compound (II) the H⋯H contacts at 42.2% (Fig. 10 ▸ *b*) make a slightly higher contribution than in (I), while the C⋯H/H⋯C contacts at 23.6% (Fig. 10 ▸ *d*) make a slightly lower contribution than in (I). The O⋯H/H⋯O contacts (Fig. 10 ▸ *c*) in both compounds are similar; 29.1% in (I) *cf*. 29.0% in (II).

## Database survey   

A search of the Cambridge Structure Database (CSD, Version 5.40, February 2019; Groom *et al.*, 2016 ▸) for similar compounds gave one hit for 1,3-bis­[(2-meth­oxy­phen­oxy)meth­yl]benzene (CSD refcode KACQEL; Bryan *et al.*, 2003 ▸) but no hits for a 1,4-derivative. In KACQEL, the central benzene ring is inclined to the outer benzene rings by 67.60 (4) and 72.68 (6)°, while the outer benzene rings are inclined to each other by 69.61 (6)°. In compound (I), the central benzene ring is inclined to the outer benzene ring(s) by 36.74 (9)°, while the outer benzene rings are inclined to each other by 59.96 (10)°. In compound (II), the corresponding dihedral angles are 89.87 (2) and 0.0°, respectively.

A search for 4-benz­yloxy-3-meth­oxy­benzaldehydes gave eight hits. Apart from 4-benz­yloxy-3-meth­oxy­benzaldehyde itself (vanillin benzyl ether: COBNUC; Gerkin, 1999 ▸), the other hits include the 4-nitro­benz­yloxy derivative (VOHYUN; Li & Chen, 2008 ▸), the 4-fluoro­benz­yloxy derivative (POMQIT; Bernard-Gauthier & Schirrmacher, 2014 ▸) and the 4-chloro­benz­yloxy derivative (WINROB; Liu *et al.*, 2007 ▸). In VOHYUN, the 3-meth­oxy­benzaldehyde ring is inclined to the 4-benz­yloxy ring by 5.00 (11)°, while in COBNUC this dihedral angle is 78.11 (9)°. In POMQIT and WINROB, the corresponding dihedral angles are 69.02 (5) and 72.59 (19)°, respectively, similar to the values observed in KACQEL, *viz*. 67.60 (4) and 72.68 (6)°.

## Synthesis and crystallization   

**Compound (I**): To vanillin (0.63 g, 4.1 mmol) dissolved in 20 ml DMF was added potassium carbonate (1.7 g, 12.5 mmol) and the mixture was stirred at room temperature followed by addition of 1,3-bis­(bromo­meth­yl)benzene (0.5 g, 1.9 mmol). The reaction was allowed to proceed for 12 h. Then the reaction mixture was partitioned between water and ethyl acetate. The ethyl acetate layer was collected and concentrated under reduced pressure. The crude product obtained was recrystallized by using ethyl acetate. Colourless block-like crystals were obtained on slow evaporation of the solvent (98%).

**Compound (II)**: To vanillin (0.63 g, 4.1 mmol) dissolved in 20 ml DMF, was added potassium carbonate (1.7 g, 12.5 mmol) and the mixture was stirred at room temperature followed by addition of 1,4-bis­(bromo­meth­yl)benzene (0.5 g, 1.9 mmol). The reaction was allowed to proceed for 12 h. After the reaction mixture was partitioned between water and ethyl acetate, the ethyl acetate layer was collected and concentrated under reduced pressure. The crude product was recrystallized by using ethyl acetate. Colourless block-like crystals were obtained on slow evaporation of the solvent (98%).

## Refinement   

Crystal data, data collection and structure refinement details are summarized in Table 3 ▸. For both compounds, the hydrogen atoms were fixed geometrically and allowed to ride on their parent atoms: C—H = 0.93–0.97 Å with *U*_iso_(H) = 1.5*U*_eq_(C-meth­yl) and 1.2*U*_eq_(N,C) for other H atoms.

## Supplementary Material

Crystal structure: contains datablock(s) global, I, II. DOI: 10.1107/S2056989019006662/su5484sup1.cif

Structure factors: contains datablock(s) I. DOI: 10.1107/S2056989019006662/su5484Isup2.hkl

Structure factors: contains datablock(s) II. DOI: 10.1107/S2056989019006662/su5484IIsup3.hkl

Supporting information file. DOI: 10.1107/S2056989019006662/su5484Isup4.cml

Supporting information file. DOI: 10.1107/S2056989019006662/su5484IIsup5.cml

CCDC references: 1571498, 1571497

Additional supporting information:  crystallographic information; 3D view; checkCIF report

## Figures and Tables

**Figure 8:**
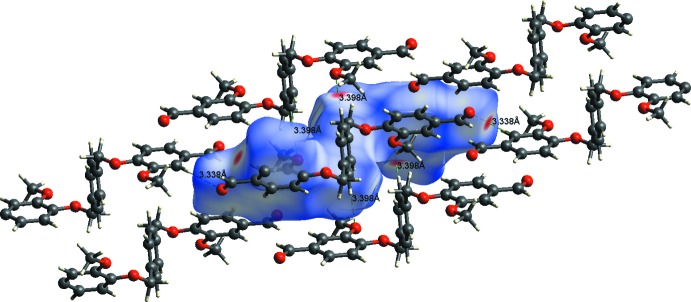
A view of the Hirshfeld surface mapped over *d*_norm_ for compound (II), showing the various inter­molecular contacts in the crystal.

**Table 1:** Hydrogen-bond geometry (Å, °) for (I) *Cg*1 is the centroid of the C2–C7 ring.

*D*—H⋯*A*	*D*—H	H⋯*A*	*D*⋯*A*	*D*—H⋯*A*
C3—H3⋯O2^i^	0.93	2.53	3.3723 (1)	151
C9—H9*B*⋯*Cg*1^ii^	0.97	2.81	3.7808 (1)	144

Symmetry codes: (i) 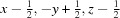; (ii) 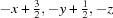.

**Table 2:** Hydrogen-bond geometry (Å, °) for (II)

*D*—H⋯*A*	*D*—H	H⋯*A*	*D*⋯*A*	*D*—H⋯*A*
C7—H7⋯O3^i^	0.93	2.47	3.338 (1)	156
C12—H12⋯O2^ii^	0.93	2.52	3.399 (1)	157

Symmetry codes: (i) 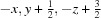; (ii) 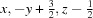.

**Table 3:** Experimental details

	(I)	(II)
Crystal data		
Chemical formula	C_24_H_22_O_6_	C_24_H_22_O_6_
*M*_r_	406.41	406.41
Crystal system, space group	Monoclinic, *C*2/*c*	Monoclinic, *P*2_1_/*c*
Temperature (K)	293	296
*a*, *b*, *c* (Å)	11.7026 (3), 14.6628 (4), 12.7512 (3)	12.6668 (5), 7.7470 (3), 10.4244 (4)
β (°)	107.863 (2)	102.126 (2)
*V* (Å^3^)	2082.54 (9)	1000.12 (7)
*Z*	4	2
Radiation type	Mo *K*α	Mo *K*α
μ (mm^−1^)	0.09	0.10
Crystal size (mm)	0.26 × 0.19 × 0.11	0.24 × 0.19 × 0.14

Data collection		
Diffractometer	Bruker SMART APEXII area detector	Bruker SMART APEXII area detector
Absorption correction	Multi-scan (*SADABS*; Bruker, 2008 ▸)	Multi-scan (*SADABS*; Bruker, 2008 ▸)
*T*_min_, *T*_max_	0.753, 0.842	0.741, 0.863
No. of measured, independent and observed [*I* > 2σ(*I*)] reflections	10081, 2595, 1497	9260, 2488, 1764
*R*_int_	0.024	0.028
(sin θ/λ)_max_ (Å^−1^)	0.668	0.670

Refinement		
*R*[*F* ^2^ > 2σ(*F* ^2^)], *wR*(*F* ^2^), *S*	0.052, 0.178, 1.05	0.057, 0.190, 1.14
No. of reflections	2595	2488
No. of parameters	138	138
H-atom treatment	H-atom parameters constrained	H-atom parameters constrained
Δρ_max_, Δρ_min_ (e Å^−3^)	0.35, −0.22	0.21, −0.21

Computer programs: *APEX2* and *SAINT* (Bruker, 2008 ▸), *SHELXS97* (Sheldrick, 2008 ▸), *SHELXL2016/4* (Sheldrick, 2015 ▸), *ORTEP-3 for Windows* (Farrugia, 2012 ▸) and *PLATON* (Spek, 2009 ▸).

## supplementary crystallographic information

### 4,4'-{[1,3-Phenylenebis(methylene)]bis(oxy)}bis(3-methoxybenzaldehyde) (I) Crystal data

**Table:**

C_24_H_22_O_6_	*F*(000) = 856
*M**_r_* = 406.41	*D*_x_ = 1.296 Mg m^−^^3^
Monoclinic, *C*2/*c*	Mo *K*α radiation, λ = 0.71073 Å
*a* = 11.7026 (3) Å	Cell parameters from 2595 reflections
*b* = 14.6628 (4) Å	θ = 2.3–28.4°
*c* = 12.7512 (3) Å	µ = 0.09 mm^−^^1^
β = 107.863 (2)°	*T* = 293 K
*V* = 2082.54 (9) Å^3^	Block, colourless
*Z* = 4	0.26 × 0.19 × 0.11 mm

### 4,4'-{[1,3-Phenylenebis(methylene)]bis(oxy)}bis(3-methoxybenzaldehyde) (I) Data collection

**Table:**

Bruker SMART APEXII area detector diffractometer	1497 reflections with *I* > 2σ(*I*)
ω and φ scans	*R*_int_ = 0.024
Absorption correction: multi-scan (*SADABS*; Bruker, 2008)	θ_max_ = 28.4°, θ_min_ = 2.3°
*T*_min_ = 0.753, *T*_max_ = 0.842	*h* = −15→15
10081 measured reflections	*k* = −13→19
2595 independent reflections	*l* = −17→16

### 4,4'-{[1,3-Phenylenebis(methylene)]bis(oxy)}bis(3-methoxybenzaldehyde) (I) Refinement

**Table:**

Refinement on *F*^2^	Primary atom site location: structure-invariant direct methods
Least-squares matrix: full	Secondary atom site location: difference Fourier map
*R*[*F*^2^ > 2σ(*F*^2^)] = 0.052	Hydrogen site location: inferred from neighbouring sites
*wR*(*F*^2^) = 0.178	H-atom parameters constrained
*S* = 1.05	*w* = 1/[σ^2^(*F*_o_^2^) + (0.0852*P*)^2^ + 0.5811*P*] where *P* = (*F*_o_^2^ + 2*F*_c_^2^)/3
2595 reflections	(Δ/σ)_max_ < 0.001
138 parameters	Δρ_max_ = 0.35 e Å^−^^3^
0 restraints	Δρ_min_ = −0.22 e Å^−^^3^

### 4,4'-{[1,3-Phenylenebis(methylene)]bis(oxy)}bis(3-methoxybenzaldehyde) (I) Special details

**Table:**

Geometry. All esds (except the esd in the dihedral angle between two l.s. planes) are estimated using the full covariance matrix. The cell esds are taken into account individually in the estimation of esds in distances, angles and torsion angles; correlations between esds in cell parameters are only used when they are defined by crystal symmetry. An approximate (isotropic) treatment of cell esds is used for estimating esds involving l.s. planes.

### 4,4'-{[1,3-Phenylenebis(methylene)]bis(oxy)}bis(3-methoxybenzaldehyde) (I) Fractional atomic coordinates and isotropic or equivalent isotropic displacement parameters (Å^2^)

**Table:**

	*x*	*y*	*z*	*U*_iso_*/*U*_eq_	
O3	0.93872 (11)	0.15775 (9)	0.04447 (9)	0.0615 (4)	
C10	0.94216 (16)	0.02577 (13)	0.15339 (13)	0.0541 (5)	
C9	0.87534 (17)	0.07592 (13)	0.05044 (14)	0.0605 (5)	
H9A	0.869121	0.038180	−0.013554	0.073*	
H9B	0.794849	0.090322	0.051631	0.073*	
C2	0.81181 (18)	0.33726 (15)	−0.21384 (14)	0.0668 (6)	
C5	0.89059 (16)	0.21460 (13)	−0.04217 (12)	0.0550 (5)	
C4	0.78416 (17)	0.19835 (14)	−0.12530 (14)	0.0632 (5)	
H4	0.739281	0.146221	−0.124167	0.076*	
C11	1.000000	0.07241 (18)	0.250000	0.0563 (6)	
H11	1.000000	0.135837	0.250002	0.068*	
O2	1.05928 (15)	0.30383 (11)	0.04307 (12)	0.0928 (6)	
C3	0.74587 (18)	0.26096 (15)	−0.20991 (14)	0.0683 (6)	
H3	0.673846	0.251044	−0.265279	0.082*	
C6	0.95715 (17)	0.29375 (14)	−0.04386 (14)	0.0648 (5)	
C12	0.94372 (17)	−0.06856 (13)	0.15437 (15)	0.0621 (5)	
H12	0.906532	−0.100685	0.090051	0.075*	
C13	1.000000	−0.1151 (2)	0.250000	0.0713 (8)	
H13	1.000002	−0.178546	0.249999	0.086*	
O1	0.81947 (19)	0.47012 (15)	−0.31880 (14)	0.1063 (7)	
C7	0.91931 (18)	0.35402 (15)	−0.12987 (15)	0.0691 (6)	
H7	0.964657	0.405581	−0.132429	0.083*	
C1	0.7698 (2)	0.4010 (2)	−0.30609 (17)	0.0851 (7)	
H1	0.698090	0.386782	−0.359846	0.102*	
C8	1.1085 (3)	0.3927 (2)	0.0642 (3)	0.1509 (18)	
H8A	1.137086	0.411446	0.004552	0.226*	
H8B	1.173946	0.392483	0.131560	0.226*	
H8C	1.047747	0.434366	0.070672	0.226*	

### 4,4'-{[1,3-Phenylenebis(methylene)]bis(oxy)}bis(3-methoxybenzaldehyde) (I) Atomic displacement parameters (Å^2^)

**Table:**

	*U*^11^	*U*^22^	*U*^33^	*U*^12^	*U*^13^	*U*^23^
O3	0.0645 (8)	0.0617 (8)	0.0401 (6)	−0.0032 (6)	−0.0109 (5)	0.0053 (5)
C10	0.0556 (10)	0.0551 (11)	0.0448 (8)	−0.0004 (8)	0.0052 (7)	−0.0028 (7)
C9	0.0664 (11)	0.0584 (12)	0.0413 (8)	−0.0049 (9)	−0.0060 (7)	−0.0057 (8)
C2	0.0714 (12)	0.0714 (14)	0.0412 (9)	0.0141 (10)	−0.0072 (8)	0.0046 (8)
C5	0.0586 (10)	0.0587 (11)	0.0342 (7)	0.0051 (9)	−0.0057 (7)	−0.0016 (7)
C4	0.0626 (11)	0.0659 (13)	0.0444 (9)	0.0019 (9)	−0.0082 (8)	−0.0051 (8)
C11	0.0637 (15)	0.0482 (15)	0.0437 (12)	0.000	−0.0031 (10)	0.000
O2	0.0826 (10)	0.0899 (12)	0.0666 (9)	−0.0222 (8)	−0.0352 (7)	0.0219 (7)
C3	0.0660 (12)	0.0761 (14)	0.0416 (9)	0.0075 (11)	−0.0145 (8)	−0.0031 (8)
C6	0.0623 (11)	0.0712 (14)	0.0420 (9)	−0.0009 (9)	−0.0119 (8)	0.0034 (8)
C12	0.0711 (12)	0.0556 (12)	0.0541 (10)	−0.0039 (9)	0.0110 (9)	−0.0113 (8)
C13	0.090 (2)	0.0497 (17)	0.0703 (17)	0.000	0.0194 (15)	0.000
O1	0.1169 (15)	0.0983 (14)	0.0792 (11)	0.0096 (11)	−0.0061 (10)	0.0360 (10)
C7	0.0709 (12)	0.0692 (13)	0.0496 (9)	−0.0004 (10)	−0.0076 (8)	0.0088 (9)
C1	0.0874 (16)	0.0919 (18)	0.0544 (11)	0.0155 (14)	−0.0100 (10)	0.0169 (11)
C8	0.156 (3)	0.125 (3)	0.102 (2)	−0.072 (2)	−0.063 (2)	0.0382 (19)

### 4,4'-{[1,3-Phenylenebis(methylene)]bis(oxy)}bis(3-methoxybenzaldehyde) (I) Geometric parameters (Å, º)

**Table:**

O3—C5	1.360 (2)	C11—H11	0.9300
O3—C9	1.425 (2)	O2—C6	1.366 (2)
C10—C12	1.383 (3)	O2—C8	1.416 (3)
C10—C11	1.390 (2)	C3—H3	0.9300
C10—C9	1.499 (2)	C6—C7	1.372 (3)
C9—H9A	0.9700	C12—C13	1.376 (2)
C9—H9B	0.9700	C12—H12	0.9300
C2—C3	1.369 (3)	C13—C12^i^	1.376 (2)
C2—C7	1.401 (3)	C13—H13	0.9300
C2—C1	1.464 (3)	O1—C1	1.204 (3)
C5—C4	1.386 (2)	C7—H7	0.9300
C5—C6	1.402 (3)	C1—H1	0.9300
C4—C3	1.382 (3)	C8—H8A	0.9600
C4—H4	0.9300	C8—H8B	0.9600
C11—C10^i^	1.390 (2)	C8—H8C	0.9600
			
C5—O3—C9	117.82 (13)	C2—C3—H3	119.3
C12—C10—C11	118.85 (17)	C4—C3—H3	119.3
C12—C10—C9	120.03 (15)	O2—C6—C7	124.50 (19)
C11—C10—C9	121.09 (17)	O2—C6—C5	115.42 (16)
O3—C9—C10	108.65 (13)	C7—C6—C5	120.07 (16)
O3—C9—H9A	110.0	C13—C12—C10	120.36 (18)
C10—C9—H9A	110.0	C13—C12—H12	119.8
O3—C9—H9B	110.0	C10—C12—H12	119.8
C10—C9—H9B	110.0	C12—C13—C12^i^	120.5 (3)
H9A—C9—H9B	108.3	C12—C13—H13	119.7
C3—C2—C7	119.97 (17)	C12^i^—C13—H13	119.7
C3—C2—C1	119.81 (18)	C6—C7—C2	119.5 (2)
C7—C2—C1	120.2 (2)	C6—C7—H7	120.3
O3—C5—C4	124.52 (18)	C2—C7—H7	120.3
O3—C5—C6	115.23 (14)	O1—C1—C2	126.1 (2)
C4—C5—C6	120.25 (16)	O1—C1—H1	117.0
C3—C4—C5	118.9 (2)	C2—C1—H1	117.0
C3—C4—H4	120.6	O2—C8—H8A	109.5
C5—C4—H4	120.6	O2—C8—H8B	109.5
C10—C11—C10^i^	121.0 (2)	H8A—C8—H8B	109.5
C10—C11—H11	119.5	O2—C8—H8C	109.5
C10^i^—C11—H11	119.5	H8A—C8—H8C	109.5
C6—O2—C8	117.20 (18)	H8B—C8—H8C	109.5
C2—C3—C4	121.33 (16)		
			
C5—O3—C9—C10	−178.26 (15)	O3—C5—C6—O2	−1.6 (3)
C12—C10—C9—O3	−145.60 (18)	C4—C5—C6—O2	178.78 (19)
C11—C10—C9—O3	36.5 (2)	O3—C5—C6—C7	177.39 (17)
C9—O3—C5—C4	−0.1 (3)	C4—C5—C6—C7	−2.2 (3)
C9—O3—C5—C6	−179.75 (17)	C11—C10—C12—C13	1.1 (3)
O3—C5—C4—C3	−178.96 (17)	C9—C10—C12—C13	−176.87 (16)
C6—C5—C4—C3	0.6 (3)	C10—C12—C13—C12^i^	−0.54 (13)
C12—C10—C11—C10^i^	−0.52 (13)	O2—C6—C7—C2	−179.0 (2)
C9—C10—C11—C10^i^	177.38 (19)	C5—C6—C7—C2	2.1 (3)
C7—C2—C3—C4	−1.2 (3)	C3—C2—C7—C6	−0.4 (3)
C1—C2—C3—C4	178.6 (2)	C1—C2—C7—C6	179.8 (2)
C5—C4—C3—C2	1.1 (3)	C3—C2—C1—O1	180.0 (3)
C8—O2—C6—C7	20.4 (4)	C7—C2—C1—O1	−0.2 (4)
C8—O2—C6—C5	−160.7 (3)		

Symmetry code: (i) −*x*+2, *y*, −*z*+1/2.

### 4,4'-{[1,3-Phenylenebis(methylene)]bis(oxy)}bis(3-methoxybenzaldehyde) (I) Hydrogen-bond geometry (Å, º)

*Cg*1 is the centroid of the C2–C7 ring.

**Table:**

*D*—H···*A*	*D*—H	H···*A*	*D*···*A*	*D*—H···*A*
C3—H3···O2^ii^	0.93	2.53	3.3723 (1)	151
C9—H9*B*···*Cg*1^iii^	0.97	2.81	3.7808 (1)	144

Symmetry codes: (ii) *x*−1/2, −*y*+1/2, *z*−1/2; (iii) −*x*+3/2, −*y*+1/2, −*z*.

### 4,4'-{[(1,4-Phenylenebis(methylene)]bis(oxy)}bis(3-methoxybenzaldehyde) (II) Crystal data

**Table:**

C_24_H_22_O_6_	*F*(000) = 428
*M**_r_* = 406.41	*D*_x_ = 1.350 Mg m^−^^3^
Monoclinic, *P*2_1_/*c*	Mo *K*α radiation, λ = 0.71073 Å
*a* = 12.6668 (5) Å	Cell parameters from 2488 reflections
*b* = 7.7470 (3) Å	θ = 1.6–28.4°
*c* = 10.4244 (4) Å	µ = 0.10 mm^−^^1^
β = 102.126 (2)°	*T* = 296 K
*V* = 1000.12 (7) Å^3^	Block, colourless
*Z* = 2	0.24 × 0.19 × 0.14 mm

### 4,4'-{[(1,4-Phenylenebis(methylene)]bis(oxy)}bis(3-methoxybenzaldehyde) (II) Data collection

**Table:**

Bruker SMART APEXII area detector diffractometer	1764 reflections with *I* > 2σ(*I*)
ω and φ scans	*R*_int_ = 0.028
Absorption correction: multi-scan (*SADABS*; Bruker, 2008)	θ_max_ = 28.4°, θ_min_ = 1.6°
*T*_min_ = 0.741, *T*_max_ = 0.863	*h* = −16→11
9260 measured reflections	*k* = −10→10
2488 independent reflections	*l* = −13→13

### 4,4'-{[(1,4-Phenylenebis(methylene)]bis(oxy)}bis(3-methoxybenzaldehyde) (II) Refinement

**Table:**

Refinement on *F*^2^	Secondary atom site location: difference Fourier map
Least-squares matrix: full	Hydrogen site location: inferred from neighbouring sites
*R*[*F*^2^ > 2σ(*F*^2^)] = 0.057	H-atom parameters constrained
*wR*(*F*^2^) = 0.190	*w* = 1/[σ^2^(*F*_o_^2^) + (0.067*P*)^2^ + 0.6516*P*] where *P* = (*F*_o_^2^ + 2*F*_c_^2^)/3
*S* = 1.14	(Δ/σ)_max_ < 0.001
2488 reflections	Δρ_max_ = 0.21 e Å^−^^3^
138 parameters	Δρ_min_ = −0.21 e Å^−^^3^
0 restraints	Extinction correction: (SHELXL-2016/4; Sheldrick, 2015), Fc^*^=kFc[1+0.001xFc^2^λ^3^/sin(2θ)]^-1/4^
Primary atom site location: structure-invariant direct methods	Extinction coefficient: 0.012 (4)

### 4,4'-{[(1,4-Phenylenebis(methylene)]bis(oxy)}bis(3-methoxybenzaldehyde) (II) Special details

**Table:**

Geometry. All esds (except the esd in the dihedral angle between two l.s. planes) are estimated using the full covariance matrix. The cell esds are taken into account individually in the estimation of esds in distances, angles and torsion angles; correlations between esds in cell parameters are only used when they are defined by crystal symmetry. An approximate (isotropic) treatment of cell esds is used for estimating esds involving l.s. planes.

### 4,4'-{[(1,4-Phenylenebis(methylene)]bis(oxy)}bis(3-methoxybenzaldehyde) (II) Fractional atomic coordinates and isotropic or equivalent isotropic displacement parameters (Å^2^)

**Table:**

	*x*	*y*	*z*	*U*_iso_*/*U*_eq_	
O2	0.30136 (14)	1.1675 (2)	0.5834 (2)	0.0541 (5)	
O1	0.36397 (14)	0.9305 (2)	0.44499 (19)	0.0523 (5)	
C6	0.23878 (19)	1.0232 (3)	0.5659 (2)	0.0420 (5)	
C5	0.27247 (18)	0.8936 (3)	0.4887 (2)	0.0418 (5)	
C7	0.14955 (19)	0.9951 (3)	0.6183 (2)	0.0443 (6)	
H7	0.127746	1.079201	0.670885	0.053*	
C4	0.2124 (2)	0.7427 (3)	0.4619 (3)	0.0477 (6)	
H4	0.233030	0.658649	0.408429	0.057*	
C2	0.09122 (19)	0.8417 (3)	0.5933 (2)	0.0450 (6)	
O3	−0.05970 (18)	0.6894 (3)	0.6395 (2)	0.0730 (7)	
C3	0.1222 (2)	0.7173 (3)	0.5146 (3)	0.0493 (6)	
H3	0.082419	0.616013	0.496873	0.059*	
C10	0.45132 (19)	0.6482 (3)	0.4372 (2)	0.0421 (5)	
C9	0.4032 (2)	0.8059 (3)	0.3651 (3)	0.0543 (7)	
H9A	0.457346	0.860256	0.325172	0.065*	
H9B	0.343984	0.770853	0.294866	0.065*	
C11	0.5138 (2)	0.6577 (3)	0.5620 (3)	0.0520 (7)	
H11	0.523957	0.763637	0.604812	0.062*	
C1	−0.0040 (2)	0.8164 (4)	0.6514 (3)	0.0582 (7)	
H1	−0.022677	0.906265	0.701458	0.070*	
C8	0.2642 (3)	1.3100 (3)	0.6471 (4)	0.0684 (9)	
H8A	0.193267	1.342179	0.600621	0.103*	
H8B	0.312586	1.405680	0.648261	0.103*	
H8C	0.261680	1.278595	0.735459	0.103*	
C12	0.4386 (2)	0.4880 (3)	0.3760 (3)	0.0526 (7)	
H12	0.397133	0.479111	0.291377	0.063*	

### 4,4'-{[(1,4-Phenylenebis(methylene)]bis(oxy)}bis(3-methoxybenzaldehyde) (II) Atomic displacement parameters (Å^2^)

**Table:**

	*U*^11^	*U*^22^	*U*^33^	*U*^12^	*U*^13^	*U*^23^
O2	0.0558 (10)	0.0340 (9)	0.0740 (13)	−0.0046 (7)	0.0172 (9)	−0.0053 (8)
O1	0.0537 (10)	0.0376 (9)	0.0721 (12)	0.0043 (8)	0.0280 (9)	0.0017 (8)
C6	0.0461 (12)	0.0313 (11)	0.0468 (13)	0.0027 (9)	0.0058 (10)	0.0020 (9)
C5	0.0441 (12)	0.0339 (11)	0.0487 (13)	0.0048 (9)	0.0128 (10)	0.0057 (9)
C7	0.0468 (12)	0.0398 (12)	0.0473 (13)	0.0049 (10)	0.0124 (10)	−0.0013 (10)
C4	0.0538 (14)	0.0333 (12)	0.0562 (15)	0.0038 (10)	0.0123 (11)	−0.0059 (10)
C2	0.0458 (12)	0.0413 (12)	0.0476 (13)	0.0020 (10)	0.0087 (10)	0.0062 (10)
O3	0.0699 (13)	0.0722 (14)	0.0826 (15)	−0.0165 (11)	0.0287 (11)	0.0099 (12)
C3	0.0470 (13)	0.0375 (12)	0.0618 (16)	−0.0029 (10)	0.0076 (11)	0.0005 (11)
C10	0.0430 (12)	0.0393 (12)	0.0491 (13)	0.0012 (9)	0.0214 (10)	−0.0006 (10)
C9	0.0628 (16)	0.0485 (14)	0.0588 (16)	0.0096 (12)	0.0293 (13)	0.0075 (12)
C11	0.0605 (15)	0.0379 (13)	0.0584 (16)	0.0008 (11)	0.0143 (12)	−0.0130 (11)
C1	0.0573 (16)	0.0614 (17)	0.0589 (16)	−0.0015 (13)	0.0188 (13)	0.0042 (13)
C8	0.0711 (19)	0.0353 (13)	0.097 (2)	0.0009 (13)	0.0129 (17)	−0.0173 (14)
C12	0.0568 (15)	0.0518 (15)	0.0462 (14)	0.0042 (12)	0.0041 (11)	−0.0081 (11)

### 4,4'-{[(1,4-Phenylenebis(methylene)]bis(oxy)}bis(3-methoxybenzaldehyde) (II) Geometric parameters (Å, º)

**Table:**

O2—C6	1.361 (3)	C3—H3	0.9300
O2—C8	1.418 (3)	C10—C11	1.375 (4)
O1—C5	1.362 (3)	C10—C12	1.390 (3)
O1—C9	1.431 (3)	C10—C9	1.495 (3)
C6—C7	1.372 (3)	C9—H9A	0.9700
C6—C5	1.408 (3)	C9—H9B	0.9700
C5—C4	1.390 (3)	C11—C12^i^	1.376 (4)
C7—C2	1.395 (3)	C11—H11	0.9300
C7—H7	0.9300	C1—H1	0.9300
C4—C3	1.382 (4)	C8—H8A	0.9600
C4—H4	0.9300	C8—H8B	0.9600
C2—C3	1.375 (4)	C8—H8C	0.9600
C2—C1	1.472 (4)	C12—C11^i^	1.376 (4)
O3—C1	1.202 (3)	C12—H12	0.9300
			
C6—O2—C8	117.4 (2)	C12—C10—C9	120.2 (2)
C5—O1—C9	118.51 (19)	O1—C9—C10	114.4 (2)
O2—C6—C7	125.6 (2)	O1—C9—H9A	108.7
O2—C6—C5	115.1 (2)	C10—C9—H9A	108.7
C7—C6—C5	119.2 (2)	O1—C9—H9B	108.7
O1—C5—C4	125.3 (2)	C10—C9—H9B	108.7
O1—C5—C6	115.0 (2)	H9A—C9—H9B	107.6
C4—C5—C6	119.7 (2)	C10—C11—C12^i^	120.6 (2)
C6—C7—C2	120.6 (2)	C10—C11—H11	119.7
C6—C7—H7	119.7	C12^i^—C11—H11	119.7
C2—C7—H7	119.7	O3—C1—C2	125.5 (3)
C3—C4—C5	120.2 (2)	O3—C1—H1	117.3
C3—C4—H4	119.9	C2—C1—H1	117.3
C5—C4—H4	119.9	O2—C8—H8A	109.5
C3—C2—C7	120.1 (2)	O2—C8—H8B	109.5
C3—C2—C1	120.9 (2)	H8A—C8—H8B	109.5
C7—C2—C1	119.0 (2)	O2—C8—H8C	109.5
C2—C3—C4	120.0 (2)	H8A—C8—H8C	109.5
C2—C3—H3	120.0	H8B—C8—H8C	109.5
C4—C3—H3	120.0	C11^i^—C12—C10	121.4 (2)
C11—C10—C12	118.0 (2)	C11^i^—C12—H12	119.3
C11—C10—C9	121.7 (2)	C10—C12—H12	119.3
			
C8—O2—C6—C7	−8.4 (4)	C6—C7—C2—C1	180.0 (2)
C8—O2—C6—C5	172.7 (2)	C7—C2—C3—C4	1.2 (4)
C9—O1—C5—C4	−0.1 (4)	C1—C2—C3—C4	−179.4 (2)
C9—O1—C5—C6	−179.6 (2)	C5—C4—C3—C2	0.3 (4)
O2—C6—C5—O1	1.2 (3)	C5—O1—C9—C10	−71.7 (3)
C7—C6—C5—O1	−177.7 (2)	C11—C10—C9—O1	−39.0 (3)
O2—C6—C5—C4	−178.3 (2)	C12—C10—C9—O1	144.6 (2)
C7—C6—C5—C4	2.8 (3)	C12—C10—C11—C12^i^	−0.5 (4)
O2—C6—C7—C2	179.8 (2)	C9—C10—C11—C12^i^	−176.9 (2)
C5—C6—C7—C2	−1.3 (3)	C3—C2—C1—O3	2.0 (4)
O1—C5—C4—C3	178.3 (2)	C7—C2—C1—O3	−178.5 (3)
C6—C5—C4—C3	−2.3 (4)	C11—C10—C12—C11^i^	0.5 (4)
C6—C7—C2—C3	−0.6 (4)	C9—C10—C12—C11^i^	177.0 (2)

Symmetry code: (i) −*x*+1, −*y*+1, −*z*+1.

### 4,4'-{[(1,4-Phenylenebis(methylene)]bis(oxy)}bis(3-methoxybenzaldehyde) (II) Hydrogen-bond geometry (Å, º)

**Table:**

*D*—H···*A*	*D*—H	H···*A*	*D*···*A*	*D*—H···*A*
C7—H7···O3^ii^	0.93	2.47	3.338 (1)	156
C12—H12···O2^iii^	0.93	2.52	3.399 (1)	157

Symmetry codes: (ii) −*x*, *y*+1/2, −*z*+3/2; (iii) *x*, −*y*+3/2, *z*−1/2.
